# 

**Published:** 1849-05

**Authors:** 


### CONTENTS

OF NO. V.

---

### ORIGINAL COMMUNICATIONS.

ESSAYS AND CASES.

Art. I.—Cholera as it appeared in Nashville. By A. H. Buchanan, M.D., in a letter to Prof. Yandell..........369

Art. II.—Some remarks on the Febrile stage of Epidemic Cholera; with the suggestion of a new theory of the propagation and spread of Cholera. By Dr. E. B. Haskins, of Clarksville, Ten.; in a letter to Prof. Yandell..........379

Art. III.—A case of Cholera, with Remarks. By W. D. Barnett, M.D., of Camden, Ark..........387

REVIEWS.

Art. IV.—A Practical Treatise on the Domestic Management and most important Diseases of Advanced Life: with an appendix, containing a series of cases illustrative of a new and successful mode of treating Lumbago and other forms of Chronic Rheumatism, Sciatica, and other Neuralgic Affections, and certain forms of Paralysis. By George E. Day, M.D., Fellow of the Royal College of Physicians, and Physician to the Western General Dispensary..........390

Art. V.—1. The British and Foreign Medico-Chirurgical Review. 2. The London Lancet, for January and February. 3. The true Pathological Nature of Cholera, and an Infallible Method of treating it. By Geo. Stuart Hawthorne, M.D. 4. On the Cryptogamous Origin of Malarious and Epidemic Fevers. By J. K. Mitchell, A.M., M.D., Professor of Practical Medicine in Jefferson Medical College..........398

### SELECTIONS FROM AMERICAN AND FOREIGN JOURNALS.

Observations on Collodion in the Treatment of Diseases of the Skin........429
Camphor and Chloroform Mixture........432
Cases of Recovery from Poisoning with Chloride of Zinc, and the suggestions of an Antidote for this Poison........434
Meteorology and Health........438
Case of Lithotomy........439
Cryptogamous origin of Fever, Cholera, &c........443
Rectum terminating in a Cul-de-sac........446
Chloroform in Mania........447
Death of Dr. Zagorsky........449
Alimentary Substances........449
Treatment of Neuralgia of the Neck of the Uterus by incision of this Organ, by M. Malgaigne........449
Comparative advantages of Lithotomy and Lithotrity........449
Sugar as an anti-Aphrodisiac........451
Nitrate of Silver in Dysentery........451
Collodion as a coating for Pills........452
Adulteration of Cream of Tartar........452

### ORIGINAL INTELLIGENCE.

Clinical Medicine in Louisville........453
University of Louisville........454
Dr. Warren on Chloroform........455
Mortality of New York in 1848........455
Cholera........456
Medical Association........456
Death of Dr. Pritchard and Dr. Townes........457
Prof. Hamilton's Fracture Tables........457
Reports of Lunatic Hospitals........458
Valedictory Addresses........459
Late Frosts........459
Death from Chloroform........459
Bowman's Practical Chemistry........460
Bellevue Hospital........460
Medical Students........460

